# A taste of things to come: Effect of temporal order of information and product experience on evaluation of healthy and sustainable plant-based products

**DOI:** 10.3389/fnut.2022.983856

**Published:** 2022-09-08

**Authors:** Marija Banovic, Anne Arvola, Kyösti Pennanen, Denisa E. Duta, Kolbrún Sveinsdóttir, Nesli Sozer, Klaus G. Grunert

**Affiliations:** ^1^MAPP Centre, Department of Management, Aarhus University, Aarhus, Denmark; ^2^VTT Technical Research Centre of Finland Ltd., Espoo, Finland; ^3^School of Marketing and Communication, University of Vaasa, Vaasa, Finland; ^4^National Institute of Research and Development for Food Bioresources IBA Bucharest, Bucharest, Romania; ^5^Matis Ltd., Reykjavík, Iceland; ^6^Faculty of Food Science and Nutrition, University of Iceland, Reykjavík, Iceland

**Keywords:** temporal order of information, product experience, tasting, plant-based products, plant protein, sustainability, health information, nutritional labeling

## Abstract

Current patterns of meat consumption are considered unsustainable. Plant-based products are presented as a solution. However, while some plant-based products thrive, others do not make the cut due to the information “framing” effect issues related to the way information is presented to the consumers. Information on the nutrition and health properties of food products are usually made available at the point of purchase, but their effect on consumer product evaluation and subsequent purchase intent can also occur later, during or after consumption. This research demonstrates that the effect of nutrition information on product evaluation and purchase intention depends on when such information is made available–before first tasting or after first tasting–and that the information interacts with the taste experience in its effect on product evaluation and subsequent purchase intent. Using three plant-based products as an example, we conducted a cross-cultural experimental sensory evaluation with temporal order of information as the main between-subject experimental condition (informed before taste vs. informed after taste vs. control condition), and product experience phase (expectation vs. experience vs. post-experience phase) and information content as within-subject conditions. Information content had two levels: lower vs. higher share of oat protein in the product (i.e., source of protein vs. high in protein). The results indicate that information generally increases consumers’ purchase intentions with information before tasting having a higher weight when compared to the condition when information was presented after tasting. Presenting the information before tasting also mitigates a drop in the evaluation of taste after tasting, observed in the two other conditions. Further, taste acts as a healthiness cue, but the direction of the inference depends on the availability of health-related information: tasting in the informed condition increased the healthiness perception, whereas tasting in the uninformed condition had the opposite effect. Giving the information before the first tasting also increased the weight of healthiness as compared to taste in the formation of purchase intentions. These findings contribute to a better understanding of the effect of temporal order of information and product tasting have on the consumers’ product evaluations of plant-based products from theoretical and managerial perspectives.

## Highlights

- Product evaluations depend on the temporal order in which information is presented.
- Information presented before product tasting results better subsequent taste perceptions.
- Information presented before product tasting changes the role of taste as a health cue.
- Information before (vs. after) tasting gives greater weight to health (vs. taste).

## Introduction

### The need for innovative products with plant-based proteins

Meat and dairy have been common and an important part of European diet over the past century mainly due to its nutritional quality (related to essential amino acids and its biological value) (1). Nevertheless, recent studies have supported the nutritional value of plant-forward diets arguing that vegetarian and vegan diets are in fact “balanced” and do meet the nutritional needs of humans when ensuring that an individual is eating a wide range of “green” plant products (such as vegetables, fruits, pulses, legumes, among others) (2–4). Further, a growing body of evidence demonstrates negative effects of meat and dairy production when compared to crop production in terms of surpassing safe limits for greenhouse gas emissions, nutrient flows, and biodiversity loss (5–7). This coupled with adverse impacts of overconsumption of meat on the environment (8, 9) and health-related risks pertaining to diabetes, cancer, blood pressure, and cardiac diseases (2, 10) brings forth the need for meat and dairy consumption to be scaled back.

To address these issues, a transition toward lower meat and dairy consumption and greater plant-based consumption is desirable (3, 11, 12). One of the routes to achieve a more sustainable and healthier diet is to provide partial meat and dairy replacement (13, 14) or complete substitution with alternative proteins from plant sources (12, 15). In this way, consumers could include several proteins in their diets by partially replacing meat and dairy with plant-based ingredients through hybrid products (11, 13) or by using alternative proteins from plants, in products such as meat analogs (16) or dairy alternatives (15).

Despite the substantial growth of meat and dairy alternatives between 2010 and 2020, doubling their market size, the market share of alternative proteins remains low in the European Union (EU), accounting for just 0.7% of the European meat market and 2.5% of the dairy market (17). To facilitate further growth of alternative proteins it is vital to improve the usage of the existing plant-based resources. Alternative plant-based proteins, such as proteins from pulses, oilseeds and cereals, are generally regarded as more environmentally friendly and healthier than conventional animal-derived proteins (9, 18, 19). However, most of the alternative plant-based proteins come from oilseeds and pulses, while cereals, such as oat, are often neglected, considered of low market relevance and mainly used for animal feed (20). This is despite the fact that cereals significantly contribute to the total EU’s plant protein supply while EU’s self-sufficiency rate is on the other hand low among oilseeds (e.g., for soya 5%) (20, 21).

Most of the companies offering substitute products containing plant proteins try to mimic meat and dairy by offering plant-based products, such as, plant-based burgers, sausages, and milk (22–24). However, they do not offer products that are not necessarily meat or dairy “look-alikes,” but can offer the same amount of protein from a plant through another type of innovative product (17). By mimicking meat and dairy products another challenge arises, namely meeting the most important success criteria for consumers, product sensory experience, and mainly flavor and texture [e.g., (25)]. If a new sustainable substitute product does not live up to consumer requirements for flavor and texture, this can lead to a market failure (6), and positioning products as substitutes encourages consumers to make comparisons of the sensory properties of the substitute with the original, which can lead to disappointment and a lack of repeat purchase (16). Finally, while some plant-based alternatives thrive, others do not make the cut due to the marketing and information “framing” effect issues related to the way that information is presented to the consumers [e.g., (26)].

Still, plant-based alternatives are the future and are already appealing to an increasing number of consumers, in addition to vegans and vegetarians (27–29). Furthermore, there is no sign that the growth of the market share of plant-based products is likely to stop (30). Therefore, it is of utmost importance to provide more alternatives and choices to consumers through products that are more sustainable, environmentally friendly, healthier, tastier, and which meet different demands, by making innovative use of existing resources, such as proteins from cereals. In developing such innovative products, it is important to integrate the development of marketing communication into the development process.

### Temporal order of information effect

Consumers may see and process information about health and nutrition characteristics before their first purchase of the product. Alternatively, they may purchase the product without being aware of this information, but may see and process it later, after they have purchased and consumed the product. Whether consumers process this information before the first purchase or after the first consumption can affect consumers’ product evaluations, which in turn will influence future purchases (31, 32). Two mechanisms can be at work here. First, when consumers are informed about health properties of the product in the pre-purchase phase, they may form expectancies not only about the healthiness of the product, but also about the sensory properties of the product, as consumers often make inferences from healthiness to taste and vice versa (33). Such expectations may in turn affect the actual perception of the sensory properties during consumption due to assimilation or contrast effects (34, 35). When consumers are exposed to such information only after the first consumption, they have already formed impressions about the sensory properties of the product that were not guided by health-related expectations. Since expectations about and perceptions of sensory properties impact purchase intention, these purchase intentions can hence be expected to differ depending on when the information is given (31) and how favorable product experience is (34). Second, the relative weight of sensory properties and information about the product in the formation of purchase intention can also differ depending the temporal order of information, which again would lead to differences in purchase intentions (36). At the first purchase, decision-making is based on expectations only, and providing health information can make the health motive more salient. After the first consumption, experience with the sensory properties of the product is available, which could make the taste motive more salient.

The question therefore is–does it make a difference when consumers get the health information and if the consumers’ perceptions will be affected differently when learning health information before first product tasting compared to when this information is learned after first product tasting? The answer to this question has important practical implications for the marketing of products with nutrition and health benefits: should the information be made salient at the point of purchase, so that consumers become more likely to see and process it in the pre-purchase phase, or should it be conveyed in a way that encourages reading and processing it at home, after the first consumption experience?

It is well documented that information can affect consumers’ food product evaluations not only before, but also after consumption (13, 26), and that information can lead to inferences across relevant product characteristics. For instance, learning that the product is locally produced before consuming it can appear to make it taste better (37). A food that is labeled as healthy may be expected and subsequently also experienced as being less tasty (33). Organic food is widely believed to taste better than conventional food and this expectations carries through to actual product experience [e.g., (38); for an overview of how extrinsic cues affect taste perception see (39)]. None of the above research, however, has investigated how temporal order of information affects consumers’ product evaluations not only before first product experience (i.e., expectations) and after subsequent product tasting (i.e., experience), but also following the post-experience tasting phase. Information provided after tasting may still lead to taste inferences, but since taste experiences have already been made, they are likely to be much smaller, if they exist at all. In addition, information given after the first tasting may have a smaller impact on future purchase intentions, because the taste motive may have higher weight in the formation of purchase intentions than the health motive once taste perceptions already exist, as people tend to have limited willingness to compromise on taste for the sake of health (40). This is important to study as consumers often acquire product information after their initial product experience, and little is known about how learning product information after product is sampled for the first time affects its evaluations later during the second sampling in the post-experience phase. The product information learned after a first product experience may have effect on the evaluation of the subsequent product experience.

Based on the above, our main exploratory premise is that the effect of health and nutrition information on consumers’ product evaluations will be contingent on the temporal order in which the information is presented. The previous research indicated that assimilation and contrasting effects occur if the information about a product is presented before or after the product experience (35). When information is presented before product experience, it can affect opinions formed during product consumption when consumers try to assimilate received information with the formed experience (31, 37). On the other hand, research shows that contrasting effect occurs when consumers are presented with nutritional and health information only after the consumption, as opinions about the product sensory properties are not guided by information and sensory properties but are contrasted against received information (41). We expect that providing health and nutrition information will have a positive impact on purchase intentions because it creates expectations about healthiness. Exposure to externally generated product frames has been shown to positively impact willingness to buy product due to the consumer trust in this information, which is considered to be reliable and thus indicative of the product utility (42). When the information precedes the first tasting, it may also lead to expectations about the taste of the product, and these expectations can be positive (or negative) depending if the taste of the product meets (or not) the formed expectation levels (37). Taste expectations may affect the actual taste experience, thus affecting also the purchase intention after the first tasting (32). On the other hand, when the product information is presented after the tasting, consumers have already formed impressions of the taste, and we would expect that the combined effect of the information and taste would increase subsequent purchase intention (31). As noted, presenting the information before or after first tasting can have an impact on the relative weight of the health and taste motives in the formation of purchase intent.

<CPS_H2 Sustainable, plant-based protein products

The evaluation of food products, and particularly new sustainable products, such as plant-based protein products, is dependent on both the information provided and consumer direct experience with the product (15, 16). The modification of the sensory properties of a product for health and sustainability reasons could produce differing effects on the product evaluations. For instance, it has been shown that products containing lower levels of plant protein compared to the ones with higher levels of plant-protein are preferred due to its lower bitterness and appealing texture (15). Further, evaluations of these sustainable products tend to be based on functional cues such as information about the product characteristics related to main ingredient (i.e., plant protein) and healthiness. However, there are only a few studies looking into the effect of this information on the subsequent cognitive and sensory evaluations of these sustainable products (41).

**FIGURE 1:**
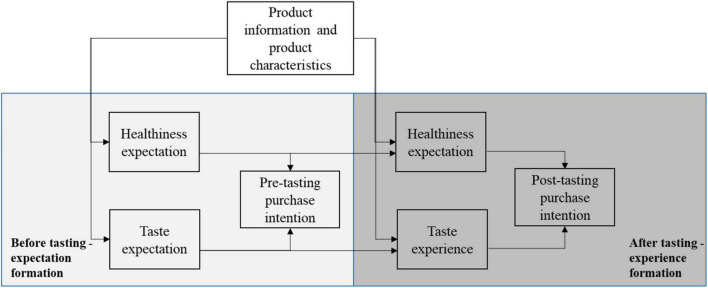
Conceptual framework.

We investigate the abovementioned research gaps and contribute to the consumer behavior and marketing literature in the following ways. We extend previous studies by exploring temporal order of information and product experience including both before and after exposure to information, as well as the post-experience phase, which is scarcely researched, but is present in real-life and important for everyday marketing practice. Food products are frequently bought, and the role that information plays in the formation of purchase intentions will change over time with repeated choices and product experiences. The effects of product information after first purchase and consumption have rarely been studied, but are of crucial importance if the aim is to encourage habits for choosing healthy and sustainable food products. Our study therefore makes a contribution to the learning that takes place over multiple purchases based on experience and information about healthy and sustainable food products. Figure 1 summarizes our conceptual framework.

## Materials and methods

### Participants and recruitment

In total, six hundred and forty three subjects were recruited across selected countries (Denmark, Finland, Iceland, and Romania) with approximately 150 subjects per country (3 groups of 50 people per experimental condition in each country). Quotas for gender and age were used in each experimental condition (i.e., 50% male; 50%, 20–40 years of age, 50%, 41–65 years of age). Participants were recruited from the general population, were all responsible for food shopping, have consumed plant-based products at least once, and were assigned to the experimental conditions beforehand following the recruitment criteria. The participants were not informed on the actual purpose of the study, but instead they were told that the study focuses on tasting of plant protein based products with oat protein enrichment. Each participant signed informed consent where the participants were informed that they are free to withdraw at any time and that the results from the study would be treated anonymously. The ethical approval for the study has been obtained both from the university’s ethical committee and the regional health institute. Sociodemographic characteristics of the participants are shown in Table 1.

**TABLE 1:** Sociodemographic characteristics of the participants.

Characteristics	Total *N* = 643	Denmark *N* = 190	Finland *N* = 150	Iceland *N* = 140	Romania *N* = 163
Age (mean) year	41.7	39.8	39.6	44.7	43.2
Gender (% female)	55.4	59.5	54.3	54.3	53.4
Marital status (%)					
- Married/co-habiting	63.0	68.4	63.3	70.7	49.4
- Single-living with parents	9.9	3.2	2.0	4.3	30.2
- Single-living independently	27.1	28.4	34.7	25.0	20.4
Children (yes, %)	49.9	47.9	50.7	48.6	52.5
Education (%)					
- Primary school	8.6	25.3	2.7	4.3	1.9
- Secondary school	13.1	11.0	20.0	20.0	1.2
- Higher education (not university)	21.0	25.3	15.3	15.7	27.8
- University (first degree, BSc)	18.8	14.7	19.3	29.3	11.7
- University (postgraduate, MSc, PhD)	38.6	23.7	42.7	30.7	57.4
Income level (%)					
- Less than average	20.7	24.7	24.7	17.9	15.4
- Average	53.0	37.9	54.6	61.4	58.0
- More than average	26.4	37.4	20.7	20.7	26.6
Consumption frequency (%)					
Plant-based products					
- Once a week and less	56.4	51.6	54.7	61.4	58.0
- 2 to 4 times a week	24.5	30.0	20.7	20.7	26.5
- 5 times a week and more	19.1	18.4	24.7	17.9	15.4

**FIGURE 2:**
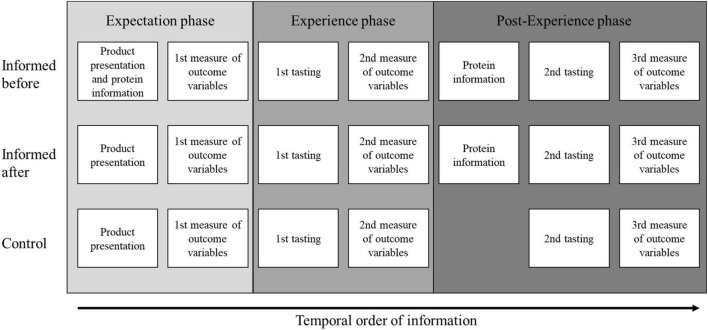
Research design.

We manipulated the order of information presentation to uncover how such information would affect subsequent product evaluations when information provided before vs. after tasting of the product, and compared to when there is no exposure to the information, only direct product experience, (i.e., control condition). We measured product evaluations three times: expectation (before tasting), experience (after 1st tasting), and post-experience (after 2nd tasting) phase. We did this for three different plant-based protein products. To ensure variation in the information provided and tasting experience, we used two versions of each of these products that were described as “source of protein” (SoP–at least 12% of the product’s energy value is provided by oat protein) and “high in protein” (HiP–at least 20% of the product’s energy value is provided by oat protein) (EC regulation No 1047/2012). The three product categories were pasta, bread, and biscuits. This procedure allowed for assessing whether the product evaluations (i.e., purchase intentions, health and taste perceptions) are affected by the temporal order in which information is presented and direct experience with the product, accounting also for the influence of product experience when more favorable vs. less favorable product experience occurs. We expected from previous research that SoP products would be evaluated more favorably than HiP products (15). Figure 2 depicts the research design of the study.

### Preparation of plant based protein-enriched products

**FIGURE 3:**
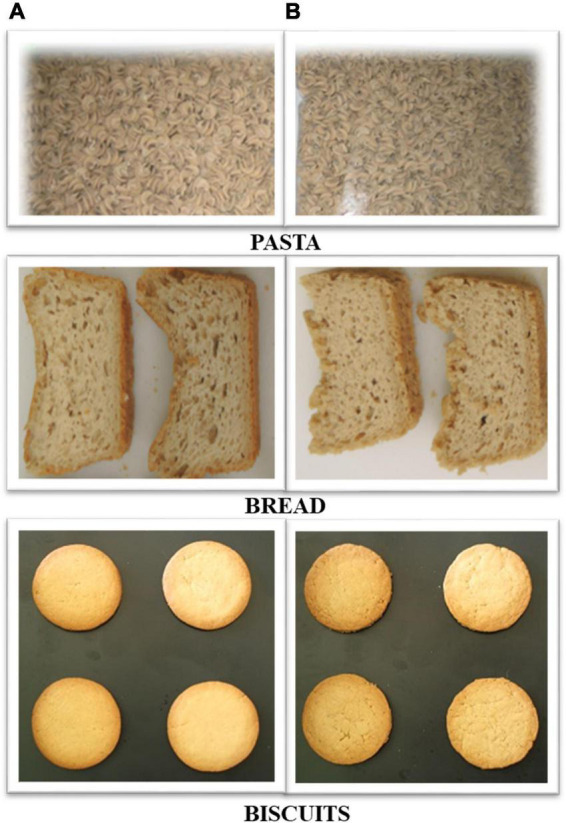
Plant-based protein enriched products: **(A)** low protein-enriched (source of protein–SoP) product, **(B)** high protein-enriched (high in protein–HiP) product.

Two different versions of protein-enriched products have been developed based on oat protein concentrate (OPC) and oat starch rich endosperm fraction (SRE) kindly provided by Fazer Mills Finland, Lahti, Finland. OPC had 28% protein, 45% starch, 5% fat and 4% dietary fiber (15) SRE had 12% protein, 60% starch, 2% fat and 8% dietary fiber. Three product categories: pasta, bread, and biscuits were produced in such a way that they could either bear the claim a “source of protein” (SoP) or the claim “high in protein” (HiP), Figure 3. We assumed that the sensory attributes of these two different product types (within each product category) would influence consumers’ perceptions and subsequent purchase intentions, and that this effect would be modified with information disclosure on protein claims on SoP and HiP enrichments, depending on the temporal order of information.

The serving portion for all products was 50 gr (i.e., one slice of bread, 1 biscuit, ∼50 g cooked pasta) per person per enrichment (i.e., 50 gr of source of protein, and 50 gr of high in protein product), thus 100 gr in total per product category.

The protein-enriched pasta was developed at pilot scale using an automatic pasta production machine (La Monferrina, Italy). Two types of spiral short pasta were obtained: OPC oat pasta (with at least 20% protein from energy value-rich in protein) from: OPC fraction, wheat semolina, modified corn starch and water in the ratio: 5:5:1:1.9 and, respectively, SRE oat pasta (with at least 12% protein from energy value-source of protein) from: SRE fraction, wheat semolina, modified corn starch and water in the ratio: 10:1.25:1:5.7. Fresh extruded pasta in the shape of fusilli (spiral short pasta) were dried for 10 h in a discontinuous dryer (La Monferrina, Italy), operating with an air flow set at 23–27°C and 56–62% relative humidity up to the final humidity around 11%. The preparation of pasta for product tasting involved cooking of pasta for 10 min in boiling water with salt (100 g dried pasta were boiled in 2.5 l water with 16 g salt), which was then rinsed with clean cold water before serving. Pasta was kept warm in covered Tupperware, which was only uncovered for the product trial.

Bread doughs were prepared by combining OPC and wheat flour. SoP bread dough had 20% OPC and 25% wheat flour whereas HiP bread dough had 38% OPC and 7% wheat flour. The ratio of remaining ingredients were the same in both bread doughs (oil 6%, sugar 1%, emulgator 0.3%, salt 0.8%, yeast 2.4% water 44%). The dry ingredients were first mixed together and then oil, yeast and water were added. The temperature of the water was adjusted so that the final dough temperature after mixing was 26 ± 1°C. Kneading was done for 120 s (slow speed) and 127 s (high speed) with a spiral mixer (Diosna SP12, Osnabrück, Germany). Baking tins were filled with 180 g of batter and then proofed for 45 min at 37°C and 70% relative humidity. The breads were baked in a rack oven (Sveba Dahlen, Fristad, Sweden) at 225°C for 20 min. One slice of bread from SoP and HiP enriched bread (about 50 gr each) was served to the participants.

Oat biscuits with SoP and HiP were prepared and provided by Fazer Bakeries (Vantaa, Finland). SoP biscuit dough had 29% OPC and 29% wheat flour whereas HiP biscuit dough had 54% OPC. The ratio of remaining ingredients were almost the same in both doughs with some small adjustments (oil 10%, sugar 9%, salt 0.5%, leavening agents 0.7%, water 24%).

All tasting sessions were held in individual sensory booths equipped with computers and online questionnaires under controlled environmental conditions with regards to light, temperature, and relative humidity. Each booth consisted of a counter top with walls that extend on three sides beyond the serving counter surface, so subjects could not view their neighbors. The subject was seated facing the computer and serving surface. Each participant was served with pair of product samples (SoP and HiP) from each of the above described product category. The two product samples were always served side by side following a Latin square or randomized order to avoid any bias in product testing. The order of two samples from each product category were also counterbalanced (43). Each sensory tasting trial was held with 10 participants.

### Design, procedure, and measures

Between-subjects experimental study has been conducted where temporal order of information has been manipulated along three experimental conditions: (i) “informed before first tasting condition,” (ii) “informed after first tasting condition,” and (iii) “control condition,” Figures 2, 5. The study also included two within-subjects factors. The first within-subject factor was *product evaluation phase*: (i) “expectation” (before tasting), (ii) “experience” (after 1st tasting), and (iii) “post-experience” (after 2nd tasting) phase. The second within-subjects factor was related to level of product protein-enrichment, namely: (i) “low-enrichment” or “source of protein” claim (SoP) and (ii) high-enrichment or “high in protein” claim (HiP) (see section “Preparation of plant based protein-enriched products”). The same experimental design has been applied across four European countries, namely Denmark, Finland, Iceland, and Romania, as well as three product categories, that is, pasta, bread, and biscuits. As indicated in Figure 5, both country and product category have been used as control variables to be able to clearly identify the relationship between independent variables (temporal order of information, between-subjects variable; product experience phase, within-subjects variable; level of product protein enrichment, within-subjects variable), and dependent variable (product evaluations), and reduce the error term (44).

In each product evaluation phase, pair of protein-enriched products, SoP (“source of protein” for low-enrichment) and HiP (“high in protein” for high-enrichment), were randomly presented two at a time from each of three products categories (pasta, bread, and biscuits), once from the beginning of the product evaluations, as indicated in Figure 2. Thus, the pair of product samples have been in front of participants the whole time while forming the expectations, experience and post-experience evaluations and filling-in the questionnaire concerning 1st, 2nd, and 3rd measure of outcome variables. The 1st, 2nd, and 3rd outcome variables were same measuring how much participants like healthiness and taste of the product with each attribute measured on a 9-point hedonic scale ranging from 1–dislike extremely; 9–like extremely (43). We also assessed at each step participants’ purchase intention on a 11-point probability scale ranging from 0–no chance, almost no chances (1 chance in 100) to certain, practically certain (99 chances in 100) (45).

**FIGURE 4:**
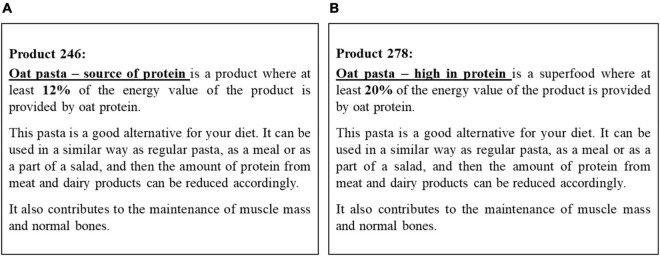
Example of information provided: Source of protein (SoP) **(A)** high inprotein (HiP) **(B)**.

As mentioned above, the order of receiving SoP and HiP product samples from three different product categories (pasta, bread, and biscuits) was counterbalanced (43). Participants filled-in separate evaluation online questionnaires for each pair of product samples from three product categories (pasta, bread and biscuits). As indicated in Figure 2, the time interval before first and the second tasting was interrupted by 2nd measure of outcome variables for all experimental groups and showing of the protein information for the informed before and informed after tasting experimental condition. In the control condition, after answering the 2nd measure of outcome variables participants were indicated to taste the two product samples again for the second time without presenting information. In the before tasting condition, each pair of products was again accompanied by their corresponding written descriptions, one saying “source of protein” (SoP for low-enrichment) and another saying “high in protein” (HiP for high-enrichment), (see example in Figure 4). In the informed after tasting condition, the participants received this information only during the post-experience phase (after second tasting).

**FIGURE 5:**
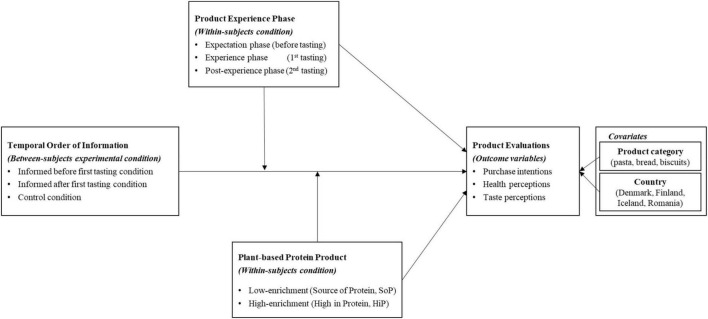
Design of repeated measures ANCOVA.

#### Informed before tasting condition

In the informed before tasting condition, at each product evaluation phase (i.e., expectation, experience, and post-experience), each pair of product samples were shown side by side always with their corresponding information on the level of protein enrichment (i.e., SoP for low and HiP for high enrichment), Figure 2. In the *expectation phase*, participants’ reported how much they like the healthiness and taste of the product (43), as well as their purchase intention (45). Subsequently, in the *experience phase*, participants repeated the same evaluations as above after having tasted each product with same information still available during tasting. Finally, in the *post-experience phase*, participants were asked to taste the products again presented along with information and evaluate them for the third time.

#### Informed after tasting condition

In the informed after tasting condition, all products were presented to the participants in the *expectation phase* without any information, only assigned numbers. The order was also randomized as mentioned above, followed by the same evaluations of measures as in the informed before tasting condition (i.e., expectations for health and taste, and purchase intention). This was followed by the *experience phase*, where blind tasting occurred, after which participants once more evaluated the product characteristics and purchase intention. At the end of the tasting, in the *post-experience phase*, participants received the full-written description of each product. After reading the full description of the products, they evaluated each product for the third time, but this time with information, in the *post-experience phase* again on the same measures, Figure 2.

#### Control condition: Blind evaluation and tasting

This condition had the same three product evaluation phases: *expectation*, *experience*, and *post-experience*. However, the participants did not receive any information about the products, and thus all evaluations and tasting were blind, Figure 2. Participants were debriefed at the end of the study.

### Data analysis

To analyze the influence of the experimental factors on the outcome variables, we ran repeated measures ANCOVA using the mixed method procedure in SPSS28, Figure 5. The analysis was performed first with purchase intention as a dependent variable, temporal order of information as main between-subjects experimental condition (i.e., informed before tasting vs. informed after tasting vs. control condition), and product evaluation phase (i.e., expectation, experience, and post-experience) and products’ protein-enrichment (i.e., low–SoP–enrichment and high–HiP–enrichment) as the within-subjects factors. We included product category type (i.e., pasta, bread and biscuits) and country (i.e., Denmark, Finland, Iceland, and Romania) as covariates, to correct for initial non-equivalences and to increase the statistical power, thus reducing the error term. We further conducted the planned contrast analysis with the Bonferroni correction to focus on a few planned comparisons between the experimental conditions and product evaluation phases that allowed us to test for the statistical significance of expected differences (46). In particular, and in relation to our assumptions in section “Temporal order of information effect,” we investigated if use of information claims on the level of protein content significantly affect product evaluations when compared to the control condition (where no information is presented), as well as if having information before product tasting vs. after product tasting significantly affects product evaluations. We repeated the above analysis by looking at the effect of temporal order of information and product experience on health and taste perceptions as outcome variables. Finally, we conducted regression analysis to explore if presenting information before vs. after first product have an impact on the relative weight of the health and taste perceptions in the formation of purchase intention (as assumed in section “Temporal order of information effect”). Therefore, we conducted separate regression analysis for each experimental condition as recommended by (47).

## Results

### The effect of temporal order of information and tasting on purchase intentions

The ANCOVA showed significant main effects for temporal order of information, evaluation phase and level of protein enrichment, as shown in Table 2. In addition, all interactions were significant, with the exception of the interaction of evaluation phase and level of protein enrichment.

**TABLE 2:** Effect of temporal order of information (experimental conditions), product evaluation phase and level of product protein-enrichment on participants’ purchase intentions, health perceptions, and taste perceptions.

Measures	Purchase intention					Health perceptions					Taste perceptions				

	*F*	*p*	η *^2^*	*95% Confidence interval*		*F*	*p*	η *^2^*	*95% Confidence interval*		*F*	*p*	η *^2^*	*95% Confidence interval*	
				Lower	Upper				Lower	Upper				Lower	Upper
*Between-subjects*															
Temporal order of information (TOF) (experimental conditions)	22.30	<0.001	0.044	0.212	0.070	0.484	0.617	0.001	0.000	0.000	0.008	0.992	0.000	0.000	0.007
*Within-subjects*															
Product evaluation phase (PEP)	3.88	0.033	0.004	0.001	0.021	13.64	<0.001	0.014	0.010	0.048	5.24	0.013	0.005	0.001	0.025
Level of product protein-enrichment (LPPE)	13.62	<0.001	0.014	0.010	0.049	5.62	0.018	0.006	0.000	0.018	55.83	<0.001	0.054	0.029	0.082
PEP × LPPE	1.17	0.301	0.001	0.000	0.005	3.29	0.047	0.003	0.000	0.009	15.76	<0.001	0.016	0.006	0.027
TOF × PEP	16.14	<0.001	0.032	0.017	0.047	11.54	<0.001	0.023	0.010	0.035	3.15	0.014	0.006	0.000	0.013
TOF × LPPE	3.60	0.028	0.007	0.000	0.010	1.63	0.196	0.003	0.000	0.006	2.63	0.072	0.005	0.000	0.008
TOF × PEP × LPPE	3.59	0.012	0.007	0.001	0.014	0.724	0.548	0.001	0.000	0.005	0.54	0.668	0.001	0.000	0.004
*Covariates*															
Country (C)	137.70	<0.001	0.124	0.088	0.162	51.53	<0.001	0.050	0.027	0.079	76.76	<0.001	0.073	0.045	0.106
Product (P)	19.24	<0.001	0.019	0.006	0.039	31.31	<0.001	0.031	0.013	0.056	6.41	0.012	0.007	0.000	0.020
TOF × Country	2.33	0.098	0.005	0.000	0.019	65.18	<0.001	0.167	0.126	0.207	34.28	<0.001	0.096	0.062	0.130
TOF × Product	1.30	0.274	0.003	0.000	0.013	22.45	<0.001	0.065	0.036	0.094	6.74	<0.001	0.020	0.005	0.039
TOF × C × P	1.45	0.234	0.003	0.000	0.014	51.03	<0.001	0.136	0.097	0.174	15.61	<0.001	0.046	0.022	0.072

**FIGURE 6:**
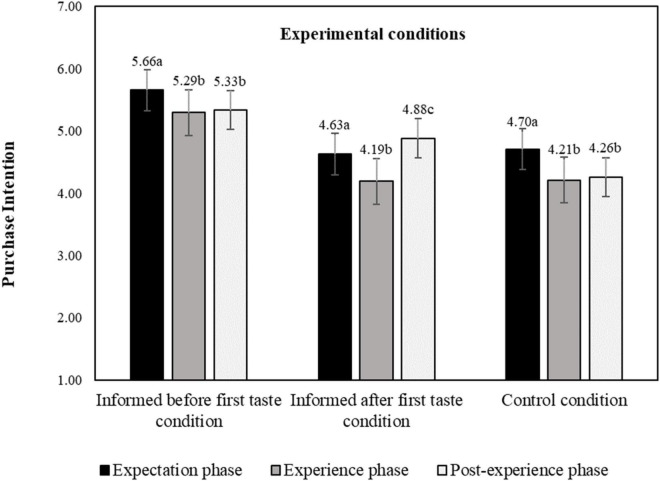
Purchase intention by product evaluation phase and experimental condition estimated, marginal means; a–c: means with different letters are significantly different at *p* < 0.05 level; purchase intention measured on a 11-point probability scale. The bars display standard errors.

Figure 6 shows purchase intention for the three experimental conditions and for the three evaluation phases. The planned contrast analysis revealed that the nutrition claims on the protein content significantly increased participants’ purchase intention compared to the control condition (*t* = 10.49, *p* < 0.001), showing a medium effect (*d* = 0.545, 95% *CI* [0.443, 0.647]). These results thus endorse the fact that participants have higher purchase intentions of plant-based products when they are informed about the protein content. Further, having information before product tasting significantly increased participants’ purchase intentions compared to having the same information presented after the product tasting (*t* = 9.75, *p* < 0.001, *d* = 0.289, 95% *CI* [0.231, 0.347]). This indicates that presenting information before tasting might have activated the health goal, giving higher weight to healthiness and lower weight to taste in the formation of purchase intention. In contrast, when participants taste first, it might give corresponding greater weight to the hedonic goal as opposed to the health goal. Alternatively, it is possible that when participants are exposed to the information first they make a cross-modal inference to a better taste and then experience it due to an assimilation effect, increasing subsequent purchase intention. We check for these assumptions in the subsequent section.

We also find that purchase intentions are significantly higher in the expectation phase than in the experience and post-experience phase, Figure 6 (except for the informed after first taste condition). This suggests that the tasting experience disconfirms the taste expectations, or, in other words, that the actual taste was not as good as participants expected and that they hence adjusted their purchase intentions downward. Also this interpretation will be checked in the following section.

**FIGURE 7:**
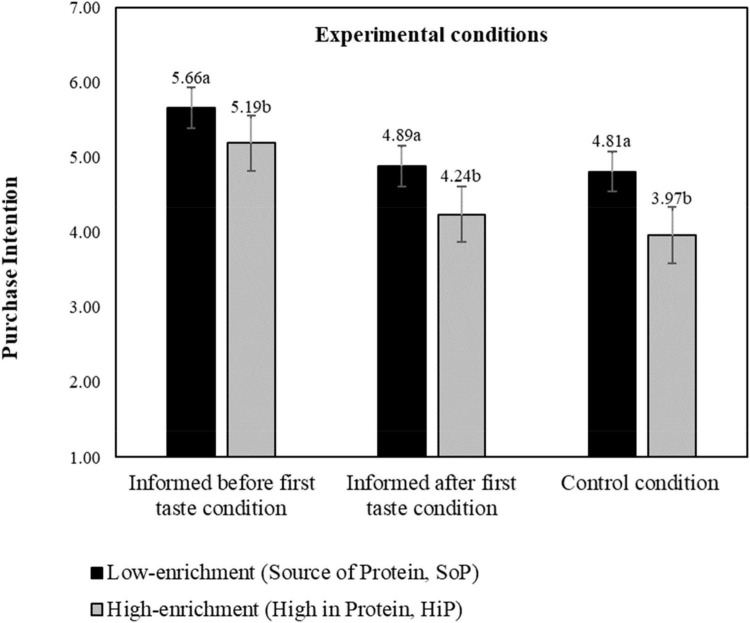
Interaction effect of experimental conditions and level of product protein-enrichment on purchase intention, estimated marginal means. a, b: means with different letters are significantly different at *p* < 0.05 level; purchase intention measured on a 11-point probability scale. The bars display standard errors.

The main effect of level of product protein-enrichment on participants’ purchase intentions was also significant (*F* = 13.62, *p* < 0.001, η^2^ = 0.014, 95% *CI* [0.010, 0.049]). As expected, the purchase intention was greater for SoP products when compared to high HiP products (*M*_*SoP*_ = 5.12, *M*_*HiP*_ = 4.47, *p* < 0.001, η^2^ = 0.115, 95% *CI* [0.080, 0.152]). We further found a significant interaction effect between temporal order of information (experimental conditions) and level of product protein-enrichment (*F* = 3.60, *p* = 0.028, η^2^ = 0.007, 95% *CI* [0.000, 0.010]). Again, we find that giving the information before the first tasting raises the purchase intention, and this goes for both levels of protein enrichment, Figure 7. We do not find significant differences between the informed after tasting and control condition (*p*_*SoP*_ = 1.00; *p*_*HiP*_ = 0.38), as expected.

### The effect of temporal order of information and tasting on health and taste perceptions

In order to shed more light on the mechanisms responsible for the effects on purchase intention found in the preceding section; we conducted additional repeated measures ANCOVAs, in which we replaced purchase intentions with health and taste perceptions as our focal outcome variables.

**FIGURE 8:**
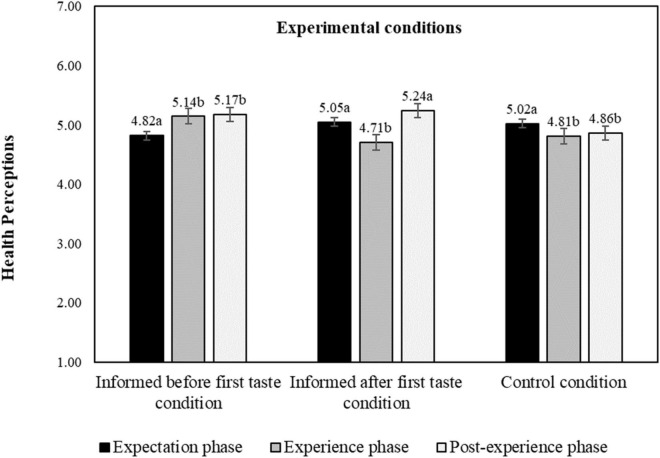
Interaction effect of experimental conditions and product evaluation phase on health perceptions, estimated marginal means. a–c: means with different letters are significantly different at *p* < 0.05 level; health perceptions measured on a 9-point hedonic scale. The bars display standard errors.

When considering health perceptions, we found that presenting information (either before or after tasting) when compared to the control condition does indeed significantly influence participants’ health perceptions (*t* = 4.48, *p* < 0.001, *d* = 0.233, 95% *CI* [0.131, 0.334]). In fact, the interaction effect of the temporal order of information with product evaluation phase shows significance (*F* = 11.54, *p* < 0.001, η^2^ = 0.023, 95% *CI* [0.010, 0.035]), Table 2 and Figure 8.

In the informed after tasting condition health perceptions decrease after first tasting (*M*_*Exp._IAFT*_ = 5.05, *M*_*Exper._IAFT*_ = 4.71, *p* < 0.001) but increase again after receiving information and second tasting (i.e., post-experience phase) (*M*_*Post–Exper._IAFT*_ = 5.24, *p* < 0.001). We thus find that health perceptions decrease after tasting when no information has been presented, but increase after tasting when information has been presented. Taste seems to work as a health cue, but the interpretation of this cue depends on the availability of the information. When there was no information on protein content, participants take the taste as an indication of a lower degree of healthiness. When, however, information about the protein content was available when tasting, the effect reverses and the taste is taken as an indicator of a higher degree of healthiness. Indeed, presenting the information alone without being able to taste does not seem to influence expectations about healthiness; it needs to be combined with the taste experience.

Furthermore, we find that SoP products are on average perceived as healthier than HiP products across all evaluation phases (*M*_*SoP*_ = 5.06, *M*_*HiP*_ = 4.90, *p* < 0.001), as we find significance main effect on health perceptions. However, we do not find significant interaction effect of level of product protein-enrichment with experimental conditions on health perceptions (*F* = 1.63, *p* = 0.196, η^2^ = 0.003, 95% *CI* [0.000, 0.006]), Table 2.

**FIGURE 9:**
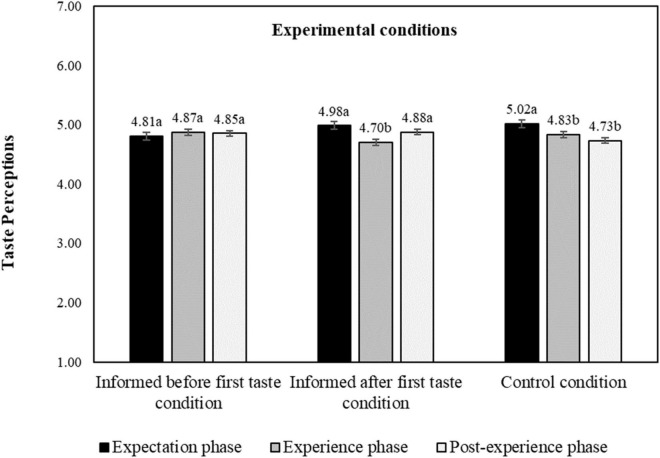
Interaction effect of experimental conditions and product evaluation phase on taste perceptions, estimated marginal means. a–c: means with different letters are significantly different at *p* < 0.05 level; taste perceptions measured on a 9-point hedonic scale. The bars display standard errors.

For the taste perceptions, both the main effect of product evaluation phase (*F* = 5.24, *p* = 0.013, η^2^ = 0.005, 95% *CI* [0.001, 0.025]) and its interaction effect with experimental conditions was significant (*F* = 3.15, *p* = 0.014, η^2^ = 0.006, 95% *CI* [0.000, 0.013]), Table 2 and Figure 9 shows that taste perception slightly decreases after the first tasting when no information was available, indicating that the taste perception did not live up to the taste expectations. Giving information about the protein content, however, mitigates this effect. When information is presented first, so that both tasting occur with information available, the taste perceptions remains at the same level after first tasting (*M*_*Exp*_. = 4.81, *M_*Exper*_.* = 4.87, *p* = 0.575), and stays constant even in the post-experience phase (*M*_*Post–Exper*_. = 4.85, *p* = 0.759). When information is given after the first tasting, the taste perception first decreases after the first tasting (*M_*Exp*_.* = 4.98, *M*_*Exper*_. = 4.70, *p* = 0.009), but then increases again after receiving the information from experience to post experience phase (*M*_*Post–exper*_. = 4.88, *p* = 0.001).

The main effect of level of product protein enrichment was also significant (*F* = 55.83, *p* < 0.001, η^2^ = 0.054, 95% *CI* [0.029, 0.082]). Generally, SoP products were preferred over HiP products in terms of taste (*M*_*SoP*_ = 4.96, *M*_*HiP*_ = 4.74, *p* < 0.001).

Finally, to check whether presenting information before (vs. after) tasting activates the health goal (vs. hedonic goal), and offers the higher weight to the healthiness perceptions (when compared to the taste perceptions) in its impact on purchase intentions, we conducted regression analysis separately for the before tasting, after tasting, and control experimental condition. In each of the three-conducted regression analysis, the purchase intention was dependent variable, while the health and taste perceptions acted as independent variables, we transformed variables and used their *Z*-scores in the analysis and bootstrapping procedure (47). When comparing results from three experimental conditions we found that presenting information before product tasting indeed increases the weight of health perceptions compared to the taste perceptions on purchase intentions (*exp(b)_*Health*_* = 1.821, *p* < 0.001, 95% *CI* [1.623, 2.044]; *exp(b)_*Taste*_* = 1.131, *p* = 0.017, 95% *CI* [1.023, 1.252]). In contrast, when presenting information after the tasting it gives the higher weight to the taste perceptions when compared to health perceptions (*exp(b)_*Taste*_* = 1.817, *p* < 0.001, 95% *CI* [1.610, 2.040]; *exp(b)_*Health*_* = 1.149, *p* < 0.001, 95% *CI* [1.322, 1.687]).

### The main and interaction effect of country and product on product evaluations

The main between-subjects effect of country and product on participants’ purchase intentions were both significant (*F* = 137.70, *p* < 0.001, η^2^ = 0.124, 95% *CI* [0.088, 0.162]; *F* = 19.24, *p* < 0.001, η^2^ = 0.019, 95% *CI* [0.006, 0.039], respectively), Table 2. The data show that on the average Romanian and Finish participants had slightly higher intention to buy plant protein enriched products (*M*_*RO*_ = 5.97; *M*_*FI*_ = 4.77) than Danish and Icelandic participants, (*M*_*DK*_ = 4.11; *M*_*ICE*_ = 4.19). With regard to the three product categories, on average participants expressed a higher intention to buy protein-enriched pasta and bread (*M*_*Pasta*_ = 5.55; *M*_*Bread*_ = 5.22) when compared to protein-enriched biscuits (*M*_*Biscuits*_ = 4.20). However, the interaction effect of country and experimental conditions, as well as product and experimental conditions, on purchase intention were both non-significant (*p* = 0.098, *p* = 0.274, respectively).

On the other hand, when looking at the health and taste perceptions, the interaction effect of country and experimental conditions was significant in both cases (all *ps* < 0.001), Table 2. Consistent with general findings data show across countries that for health perceptions and when comparing informed after and before tasting condition health perceptions decline when no information is presented, yet rise after tasting when information is presented. This effect seems to be significantly more pronounced (all *ps* < 0.001) among Romanian and German participants (after tasting condition: *M*_*RO_ IAFT*_ = 6.81; *M*_*DK_ IAFT*_ = 6.26; before tasting condition: *M*_*RO_ IBFT*_ = 7.76; *M*_*DK_ IBFT*_ = 6.93) than Danish and Icelandic participants, (after tasting condition: *M*_*DK_ IAFT*_ = 5.06; *M*_*ICE_ IAFT*_ = 6.16; before tasting condition: *M*_*DK_ IBFT*_ = 4.92; *M*_*ICE_ IBFT*_ = 5.26). This shows that there were some differences between the countries with regards to the influence of the information given, which was certainly more effective among Romanian participants. Nevertheless, same general effect was observed across countries.

For the taste perceptions, in the informed after the first tasting condition, across all countries taste perception slightly decreases when compared to informed before first tasting condition, indicating that the taste experience was not at the same level as taste expectations. This general effect was again similarly as above more evident (all *ps* < 0.001) among Romanian and German participants (after tasting condition: *M*_*RO_ IAFT*_ = 6.39; *M*_*DK_ IAFT*_ = 5.19; before tasting condition: *M*_*RO_ IBFT*_ = 6.55; *M*_*DK_ IBFT*_ = 5.50) than Danish and Icelandic participants, (after tasting condition: *M*_*DK_ IAFT*_ = 4.34; *M*_*ICE_ IAFT*_ = 5.03; before tasting condition: *M*_*DK_ IBFT*_ = 4.66; *M*_*ICE_ IBFT*_ = 5.20). This could be due to the fact that Scandinavian participants are more accustomed to the information on protein enrichment and plant-based products which are more available on their food market (48), and thus it might be that the presented information does not have the same first-impression impact.

## Discussion

Nutrition and health claims are major means of informing consumers about characteristics of food products and are also important regarding the promotion of healthy and sustainable plant-based products (49). Research has shown, though, that consumer attention to nutrition and health claims at the point of purchase is limited (50). However, nutrition and health information can have an effect on consumers also after the purchase (32). Consumers may be more at ease with reading this type of information at home, after they have purchased the product, and possibly after the first tasting experience, but not much is presently known about the temporal effect of nutrition and health information on consumers’ product perceptions and intentions. Our study contributes to understanding how the effect of nutrition and health information on consumers’ purchase intentions, taste evaluations and health perceptions of plant-based food products differs depending on when it is presented to consumers–before the first tasting or after the first tasting. Understanding such effects is important as food products are frequently bought and most purchase intentions and product perceptions are formed for products that have been previously bought and tasted.

The main results of our study show that it does indeed make a difference when the nutrition and health information is presented [cf. (31, 32)]. Starting with the purchase intentions, we found that the effect of information is highest when it is presented before the first tasting in expectation, experience, and post-experience phases. Further, our results show that the information before tasting increases the weight of health perceptions over the taste perceptions in explaining purchase intentions. The latter is most likely due to the fact that presenting the information increases the salience of health and consequently makes health having a larger impact on the formation of purchase intentions relative to taste [cf. (33)].

Interestingly, the same type of information effect was not detected in consumers’ taste perceptions. The taste expectations before the actual tasting were similar regardless of the information presence. However, in the control condition without any information, the taste evaluations significantly dropped in contrast to the pre-tasting expectations and remained lower until the final post-experience phase evaluation. In the “informed after first tasting” condition, the taste perceptions dropped in the first blind tasting in contrast to the expectations, but bounced back in the final evaluation with the information. In the informed condition, the taste perceptions remained stable throughout all three evaluation phases. These results are aligned with the earlier findings that information in general has influence on the taste perceptions (39) and relevant and value adding information tends to enhance the taste perceptions (37), demonstrated also in the context of information related to oat-enriched foods (15). However, what is interesting here is that the information had significant effect on the taste perceptions after the first blind exposure to the products’ sensory qualities (‘informed after first taste’ condition). This might be because of the congruence between the product type and the health information. It has been found that the health information can have positive effect on the hedonic ratings especially in products (e.g., bread and pasta), which inherently carry health meanings to consumers (42, 51).

In addition, our study provides interesting new insights into how health and how nutrition and health information affects both health and taste perceptions, and on how this effect is dependent on actual taste experience. It is well-known that consumers can form subjective links between healthiness and taste, usually implying that healthy products are believed to be less tasty and vice-versa (33), even though this relationship seems to depend on a range of other factors such as the product type (42). Little is known, though, about how information and taste experience interact in the formation of health and taste experiences. Our study shows that the taste experience can indeed serve as a health cue, but that the type of inference made depends on the information provided. When no information was provided before tasting, the worse-than-expected taste was taken as a cue to indicate a worse-than-expected healthiness. However, when information about the protein content of the product was available, this effect reversed and participants increased their perception of healthiness because of the taste experience.

Our results, if corroborated by future studies, have interesting implications for the promotion of plant-based products. First, it is clear that making information available before the first tasting, i.e., at the point of purchase, is crucial. This makes the health aspect more salient in the formation of purchase intentions, raises purchase intentions, and also mitigates disappointment during the first tasting. However, the results also show that an important second effect of the information occurs during tasting. Tasting in an informed condition not only improves the taste experience, but also strengthens the health perception, by making the taste a positive health cue. This, in turn can to some extent counteract the lesser weight of healthiness as compared to taste in the post-tasting formation of purchase intentions. The boundary conditions for this to occur still need to be investigated, but a better understanding of these processes is crucial for the formation of habits in the purchase of healthy and sustainable food products.

Further, our results indicate that consumers evaluated the products with lower level of protein enrichment (i.e., SoP) tastier and healthier in comparison to the products with higher level of protein enrichment (i.e., HiP). In addition, consumers showed higher purchase intentions toward the products with less protein. There might be several reasons for this. First of all, the higher perceptions and intentions toward SoP products might be explained by the target products. Previous research indicates the importance of the fit between the carrier product and the added ingredient (52), also in the added protein domain (53). It might be that bread, pasta and biscuits were considered suitable carrier products for lower amount of protein (SoP), but not for the high amount (HiP) making the lower level of protein more favorable. Another potential explanation might be related to the protein source. Familiarity with the oat as ingredient in food has been found to influence consumers’ responses toward the products (54). As oat is not a widely known cereal for human consumption in many countries except for the Northern Europe, the stated high level of an unknown ingredient in a food might have reduced the consumers’ intentions on the products.

When it comes to the higher hedonic experience with the SoP products in contrast to HiP, the product features have a role to play. For instance, good quality pasta is defined as having high degree of firmness and elasticity (55). Proper evaluation of pasta cooking quality requires consideration of a number of factors including elasticity, firmness, surface stickiness, cooking tolerance, water absorption, and loss of solids to cooking water but also attributes related with the consumers’ acceptance: color, flavor (unusual flavor or off-flavor), palatability. Short spiral pasta obtained from SRE (SoP) had a protein content around 12%, lower than short spiral pasta obtained from OPC (HiP) with 22% protein content. The fat content of the pasta samples was in the range between 0.8 and 3%, with a higher content for HiP. HiP dried sample was darker than SoP. The addition of oat protein concentrate produced an increase in hardness and chewiness of pasta in HiP sample. Addition of protein concentrates from oat had a great impact on the pasta color, increased hardness and decreased elasticity. Chewiness and sourness increased slightly [all results on product qualities are reported in Duta et al. (55)]. Taking together, the sensory properties of the HiP products have been inferior in comparison to the SoP products potentially contributing to the respective taste evaluations.

### Limitations

Our study is based on three plant-based products (i.e., pasta, bread, and biscuits) and conducted in four European countries (Denmark, Finland, Iceland, and Romania), which strengthens the generalizability of the results. Still, the results are obviously specific to the three products investigated, the specific type of claim studied, namely a nutrition claims regarding the protein content, i.e., SoP and HiP, and oat as an protein ingredient. Further, there was no actual purchase involved and the two tastings were condensed in a short time span in a controlled lab condition. In this way, the study context is removed from the daily situation where people shop, eat, shop again, and eat again. Future studies, could investigate effect of nutrition claims in a real-life context to confirm the influence of temporal order of information and product experience on consumers’ evaluation of plant-based products and thus supplement the present study allowing for nutrition and health regulations to further evolve. In addition, since the products tested were all cereals, it would be valuable to investigate temporal-order effects using other plant-based products (e.g., meat or dairy substitutes). Grasso et al. (41) and (15) found an increase in overall liking for plant-based burgers and yogurts within informed-tasting conditions, relative to blind-tasting conditions. Thus, temporal order effects may likely extend beyond the plant-based substitutes examined here. Future research could also extend the investigation to other sensory features, such as a product’s visual appearance, texture, and bitterness.

### Practical implications

In terms of promoting the purchase and consumption of plant-based products marketed considering their content or ingredients (e.g., protein content), our results clearly emphasize the need to make the information about the nutritional properties of these products available both at the point of purchase and during consumption. While making the information available at the point of purchase has received plenty of attention and labeling mechanisms have been widely discussed, much less is known about how to make the information available again at the time of consumption. The packaging of the products is an obvious channel of communication also in the home, but other channels are conceivable, for example in the context of recipes and food blogs.

## Conclusion

Overconsumption of meat is threatening both the environment and human health, which has led to development of healthy and sustainable plant-based products. Along with the product development, informing consumers about the products and their versatile benefits is of importance to facilitate transition from meat-based diets to plant-based ones. This study analyzed the effect of temporal order of information on consumers’ purchase intentions, taste experiences and health perceptions toward oat-enriched pasta, bread, and biscuits. The results of the study showed that receiving health and nutrition related information about the products before the actual experience with the products increased all evaluations. However, the results indicate that informing consumers also after the first experience with the product leads to elevated consumers’ evaluations and experiences with the products afterward. To conclude, the study provides understanding for food companies and marketers about the importance of informing consumers before the actual product experience but also afterward. Informing consumers after the first exposure, for example through a product label, can have significant effects on the subsequent purchase intentions and product evaluations.

## Data availability statement

The datasets presented in this article are not readily available because of legal and privacy issues related to the confidentiality of the tested products, which impose limitations and impede the availability of the whole dataset. Requests to access the “minimal dataset” that is underlying the findings described and used to reach the conclusions of the manuscript are available to any qualified researchers and should be directed to MB, maba@mgmt.au.dk.

## Ethics statement

The studies involving human participants were reviewed and approved by the Aarhus University, Cognition and Behavior Lab. The patients/participants provided their written informed consent to participate in this study. The research was conducted in accordance with the ethical principles outlined in the Declaration of Helsinki.

## Author contributions

MB: conceptualization, research design, methodology, investigation, experimental lab work, formal data analysis, and writing – review and editing. AA and KP: methodology, investigation, experimental lab work, and writing – review and editing. DD and KS: experimental lab work and writing – review and editing. NS: reviewing and editing and funding. KG: conceptualization, research design, methodology, and writing – review and editing. All authors contributed to the article and approved the submitted version.
